# Antegrade embolization of varicocele with cyanoacrylate glue: a case report

**DOI:** 10.1186/s42155-024-00446-6

**Published:** 2024-06-27

**Authors:** Thomas Le Tat, Raphaël Jost, Clément Hanotin, Alexandre Lucas, Liess Laouisset, Antoine Hakime, Viseth Kuoch

**Affiliations:** 1 Service de radiologie interventionnelle, Centre Hospitalier Sud Francilien, 40 Avenue Serge Dassault, 91100 Corbeil-Essonnes, France; 2 Service de radiologie diagnostique et interventionnelle, Hôpital d’Instruction des Armées Percy, 2 Rue Lieutenant Raoul Batany, 92140 Clamart, France; 3 Service de radiologie interventionnelle, American Hospital of Paris, 55 Boulevard du Château, 92200 Neuilly-sur-Seine, France

**Keywords:** Veinous intervention, Interventional radiology, Varicocele, Embolization, Cyanoacrylate glue

## Abstract

**Background:**

Varicocele embolization is an effective, minimally invasive treatment option, with a symptom improvement rate of around 90%. However, anatomical variations and post-embolization recurrences pose challenges to its efficacy. This article discusses the antegrade embolization technique as a viable alternative for cases in which retrograde embolization fails, offering a broader spectrum of treatment options for varicocele.

**Case presentation:**

This case report details the treatment of a 27-year-old male with a left varicocele, diagnosed during infertility assessment, using an alternative embolization technique. Despite initial failed attempts at retrograde catheterization via the femoral vein, a direct inguinal puncture of the left testicular vein was successfully performed under ultrasound guidance. A mixture of Glubran® and Lipiodol® was used for embolization, achieving varicocele embolization without complications. The patient was discharged 2 hours post-procedure, with follow-up confirming the procedure’s effectiveness and safety.

**Conclusion:**

This article introduces a less invasive, ultrasound-guided technique for varicocele embolization, presenting a viable alternative to surgery when conventional retrograde methods fail.

**Graphical Abstract:**

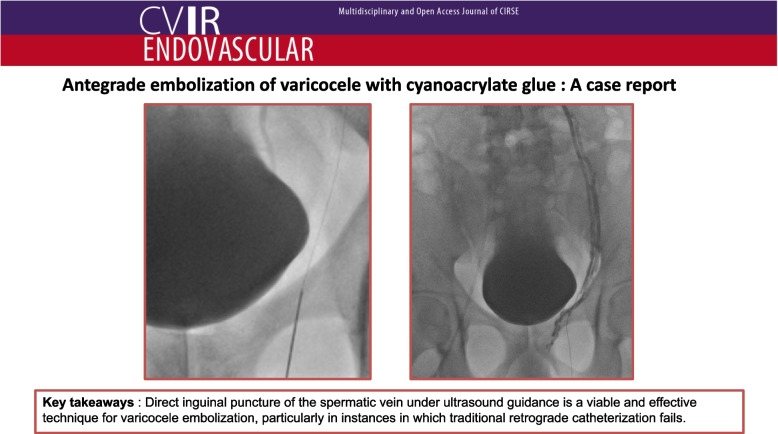

## Background

Varicocele embolization has established itself as the primary modality of treatment over the past several years, with the retrograde endovascular technique via femoral or jugular access being the predominant method employed in interventional radiology [1]. This approach boasts a high technical success rate, consistently reported in the vicinity of 90% [2]. Nonetheless, certain challenges can impede the success of this method, particularly when anatomical variations, such as a circum-aortic left renal vein, hinder the catheterization of the testicular vein. Similarly, cases of recurrence following an initial embolization and the presence of non-navigable valves present significant obstacles. Traditionally, surgical intervention has been the recourse for cases in which endovascular attempts falter. This article details an anterograde embolization technique as an effective option for managing patients for whom the retrograde approach proves ineffective, broadening the scope of treatment strategies for varicocele.

## Case presentation

In this case report, we describe the management of a 27-year-old male patient who was referred to our interventional radiology department for treatment of a left varicocele, which was identified during an evaluation for infertility. The diagnostic workup, including a spermogram, revealed oligoasthenoteratozoospermia, and ultrasound confirmed a grade III varicocele. Notably, the patient did not experience any testicular pain.

The treatment procedure was conducted under local anesthesia. The conventional retrograde access was attempted with a 6 French Launcher® Guiding Catheter – EBU 3.0 (Medtronic, Dublin, Ireland). There were no difficulties in accessing the left renal vein, nor in positioning the catheter at the junction of the spermatic vein. However, neither the guidewire nor the catheter was able to pass the valve.

**Fig. 1:**
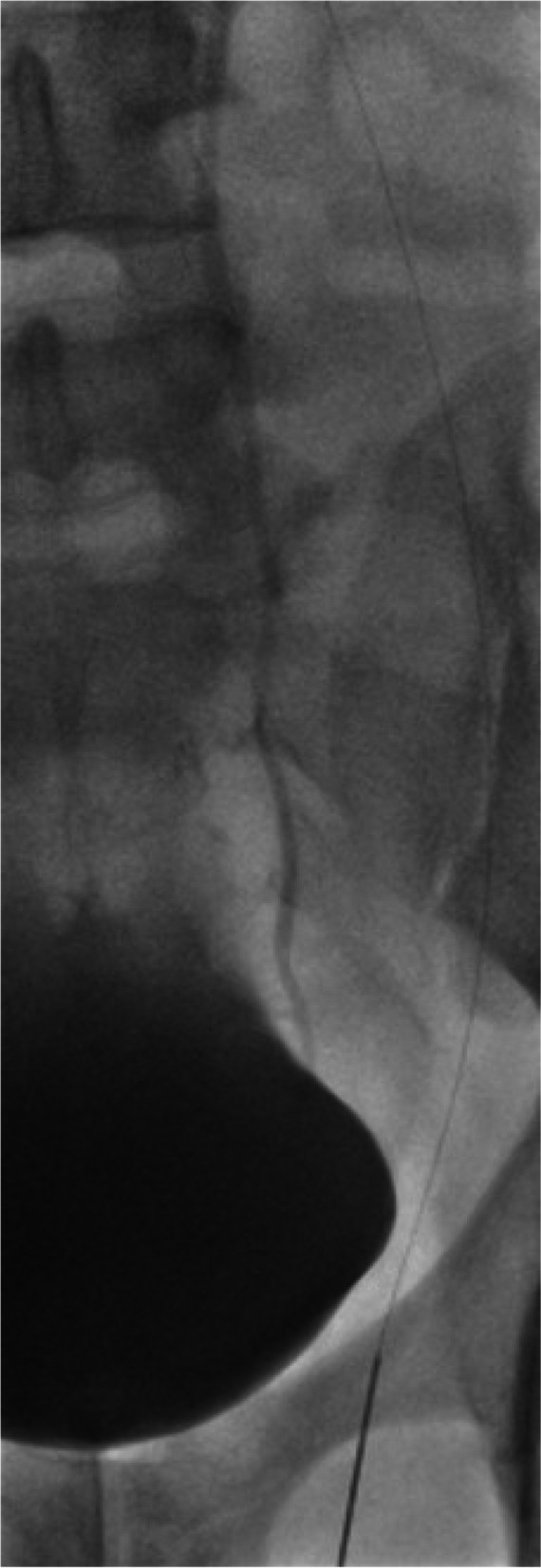
Direct puncture of the left testicular vein and catheterization with the V-18 guidewire

Consequently, we employed a substitute approach by directly puncturing the left testicular vein inguinally using a 20G spinal needle, guided by ultrasound, during a Valsalva maneuver. Following the successful puncture, a V-18® ControlWire® guidewire (Boston Scientific, United States) was introduced through the spinal needle and maneuvered until it reached the confluence of the left testicular vein and the left renal vein (Fig. 1*Direct* puncture of the left testicular vein and catheterization with the V-18 guidewire. Subsequently, the needle was carefully removed, and a Progreat® 2.7 catheter (Terumo, Japan) was inserted over the guidewire. The catheter’s tip was precisely positioned 3 cm below the junction with the left renal vein, facilitating targeted embolization.

**Fig. 2:**
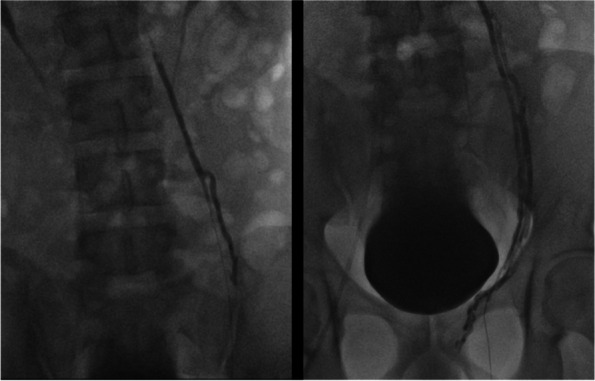
Glubran® embolization of the left testicular vein

The embolization procedure was performed using a mixture of 1 mL of Glubran® (GEM, Italy) and 2 mL of Lipiodol® (Guerbet, France), achieving successful occlusion of the varicocele (Fig. 2 Glubran® embolization of the left testicular vein.). To prevent embolization of the pampiniform plexus, manual compression was applied just above the pampiniform plexus during the embolization. Manual compression was performed by the patient to reduce radiation to the operator’s hand. Remarkably, the patient was able to be discharged merely 2 hours post-procedure without experiencing any complications. Follow-up at 3 months reported no adverse effects, and his spermogram at 3 months was improved compared with the pre-embolization spermogram.

## Discussion

The technique of varicocele treatment utilizing a sclerosing agent through the anterograde route, historically documented and executed by urological surgeons, demonstrates favorable outcomes [3]. Traditionally, this method involves an incision at the scrotal root, opening of the spermatic cord, puncturing of the testicular vein, followed by the injection of a sclerosing agent under fluoroscopic guidance. An alternative, less invasive method is achieved through the ultrasound-guided puncture of the testicular vein within the inguinal canal, yielding comparable results.

In the extant literature, only two instances of anterograde embolization following the ultrasound-guided puncture of the testicular vein are documented [4, 5]. Our case marks a pioneering instance of anterograde embolization utilizing cyanoacrylate glue via a microcatheter. The employment of a liquid embolic agent such as glue rather than coils facilitates the addressal of collateral veins, which are often implicated in the recurrence of varicocele [6–9]. Furthermore, as the glue resorbs over time, future images of the patient will not show any artifacts, unlike those obtained after coiling. The use of a microcatheter enables the precise delivery of the embolic agent at a safe distance from the scrotum, thus mitigating the risk of reflux into the scrotal veins.

However, embolization of the testicular vein with glue is not without risk. As with retrograde embolization, there is a risk of migration of the glue into the renal vein or pulmonary arteries. In our case, the contrast medium stagnated in the left testicular vein. A Valsalva maneuver combined with slow injection of the adhesive prevented the adhesive from migrating. If the patient is unable to perform a Valsalva maneuver, for example during an operation under general anesthetic, or if there is too much flow in the testicular vein, the use of a detachable coil in the proximal part of the testicular vein may allow the procedure to be performed more safely.

In addition, direct puncture of the left testicular vein can be difficult, particularly in low-grade varicocele, or in overweight patients. To facilitate direct puncture, this should be performed during a Valsalva maneuver, which requires good cooperation from the patient. Given that retrograde approach failures are relatively rare, we do not yet have enough cases of antegrade approach puncture to be able to assess the technical success rate of this procedure. A retrospective study would be interesting in the future to better measure the technical and clinical success of this procedure.

## Conclusion

This case underscores the utility of direct inguinal puncture of the testicular vein under ultrasound guidance as a viable and effective technique for varicocele embolization, particularly in instances in which traditional retrograde catheterization fails. This method not only ensures precise targeting and treatment but also highlights the potential for rapid recovery and minimal post-procedural complications, contributing significantly to the expanding arsenal of techniques in interventional radiology for the management of varicoceles.

## Data Availability

Not applicable.
